# The Sinus Tract Opening Count and Recorded Infection Control in Hip and Knee Prosthetic Joint Infection

**DOI:** 10.3390/jcm15218702

**Published:** 2026-11-07

**Authors:** Wei-Song Zhang, Lin Liu, Zhi Yang, Bin-Fei Zhang

**Affiliations:** Department of Joint Surgery, Honghui Hospital, Xi’an Jiaotong University, No. 555 Youyi East Road, Xi’an 710054, China; zhangweisongz@126.com (W.-S.Z.); liulin183092@163.com (L.L.); hhyy_yangzhi@163.com (Z.Y.)

**Keywords:** prosthetic joint infection, sinus opening count, infection control, retrospective cohort, Firth regression

## Abstract

**Background/Objectives:** A sinus tract communicating with the prosthesis is a diagnostic criterion for periprosthetic joint infection (PJI). This study investigated the association between sinus tract opening count and recorded infection control in hip and knee PJI. **Methods:** We retrospectively reviewed hospital records. Firth and conventional logistic regression analyses were adjusted for treatment strategy and preoperative microbiological specimen submission. Secondary analyses included grouped counts, joint-site interaction, sensitivity analyses, propensity-score matching, and multiple-testing correction. **Results:** Among 189 patients, 111 had knee PJI and 78 had hip PJI. Sinus openings were present in 73 patients, including 14 with multiple openings. The numbers of patients with 1, 2, 3, and 5 openings were 59, 9, 4, and 1, respectively. Infection control was recorded in 166 patients (87.8%), with 23 failures (12.2%). Control proportions in the no-opening, single-opening, and multiple-opening groups were 91.4%, 86.4%, and 64.3%. For each additional opening, the adjusted odds ratio (OR) for infection control was 0.562 (95% confidence interval [CI]: 0.340–0.912; nominal *p* = 0.0209; Bonferroni-adjusted *p* = 0.1043). The conventional logistic estimate was similar (OR 0.543, 95% CI: 0.325–0.906; *p* = 0.0195). Compared with no opening, the adjusted OR for multiple openings was 0.167 (95% CI: 0.043–0.665; *p* = 0.0121). The comparison involving multiple sinus tract openings was exploratory and hypothesis-generating, given the small number of patients in this subgroup and the multiplicity-adjusted results. **Conclusions:** In patients with PJI, an increased preoperative sinus tract opening count may be associated with lower odds of recorded infection control. Future multicenter, prospective studies with standardized counting and uniform outcome definitions are needed to validate these findings.

## 1. Introduction

In current diagnostic criteria for periprosthetic joint infection (PJI), a sinus tract communicating with the prosthesis is one of the diagnostic criteria [1,2]. After PJI is diagnosed, orthopedic surgeons also need to assess the local skin condition around the joint to determine the difficulty of subsequent treatment. A sinus tract can be detected by physical examination and is usually recorded in the medical record system as the presence or absence of sinus tract formation, the number of sinus tracts, and the presence or absence of discharge from the sinus opening.

Previous studies have explored the relationship between preoperative sinus tracts and the prognosis of patients with PJI. Xu et al. reported that the presence of a preoperative sinus tract was associated with treatment failure after two-stage revision [3]. In a study on debridement, antibiotics, and implant retention (DAIR), Deng et al. reported that the failure rates in patients with and without a sinus tract were 36.5% and 27.3%, respectively; however, the difference in survival comparisons did not reach statistical significance [4]. In addition, Luger et al. found that a sinus tract was associated with a lower infection-free survival rate [5]. These studies suggest that a preoperative sinus tract is associated with poorer prognosis, although the results are not entirely consistent.

Notably, sinus tracts vary in presentation and number: most patients have a single sinus tract, whereas a minority of patients with PJI have multiple openings. Such sinus tracts are often associated with long-standing infection, in which case local soft tissue conditions are often poor. Therefore, in addition to the presence of a sinus tract, the number of sinus tract openings is also worth investigating. However, studies systematically evaluating the number of sinus tract openings in patients with PJI are still lacking. Based on prior PJI research, we propose a possible clinical hypothesis: multiple sinus tract openings may indicate more extensive local soft tissue damage and are therefore associated with poorer infection control and prognosis. Assessing the number of openings together with the presence or absence of a sinus tract may help determine patient prognosis.

Therefore, the primary objective of this study was to investigate the association between the number of sinus tract openings and recorded infection control in PJI. The secondary objectives were to compare clinically meaningful groupings of opening counts to assess whether this association differs by joint site, and to examine the count association after accounting for sinus presence.

## 2. Materials and Methods

### 2.1. Study Design and Patient Selection

In this retrospective cohort study, we searched records of adult patients with PJI at Xi’an Honghui Hospital from 2013 to 2021. The hospital manages hip and knee arthroplasty and PJI through surgical and nonsurgical care. The study period corresponded to the years represented in the available database. The Ethics Committee of Honghui Hospital, Xi’an Jiaotong University approved this retrospective study (No. 202405014), and the Institutional Review Boards of Honghui Hospital waived the requirement for participants’ informed consent because of the study’s retrospective nature. This study was reported following the STROBE guidance [6].

We obtained demographic and clinical data for patients with PJI from their original medical records. We included adult patients (≥18 years) with a clinical diagnosis of hip or knee PJI documented by the treating team. In addition, medical records had to contain information on the presence of a sinus tract and the number of sinus tract openings, have available infection outcome records, and indicate that the patient was alive. We excluded patients with missing infection outcomes, missing records on the presence of a sinus tract or the number of openings, or death. Patient selection was based on existing medical records; the sample comprised records meeting the above inclusion and exclusion criteria, and no prospective sample size calculation was performed.

### 2.2. Hospital Treatment

Upon admission, patients underwent routine examinations. For patients undergoing preoperative microbiological testing, antibiotics were temporarily discontinued before ultrasound-guided joint aspiration. Aspirated fluid was submitted for microbiological culture and/or next-generation sequencing (NGS). Samples were not obtained directly from sinus openings. In patients undergoing surgery without a preoperatively identified pathogen, intraoperative samples were obtained for further analysis. The treatment plan was then determined based on the patient’s overall condition, the general status of the infection site, and the microbiological findings available.

All treatments analyzed in this study were delivered as part of routine clinical care before the retrospective analysis. Treatment categories included antibiotic therapy alone, one-stage revision, two-stage revision, debridement, antibiotics, and implant retention (DAIR), debridement with irrigation, amputation, and permanent spacer retention. The spacer category refers to retention of the spacer as the definitive treatment rather than as a planned interim measure before second-stage implantation. Treatment strategy was included in the model as an unordered categorical covariate to account for differences in treatment modalities recorded. These categories reflect real-world practice: because some patients were initially treated in trauma and orthopedic departments, a subset received conservative antibiotic therapy or debridement with irrigation; some patients required amputation because of severe infection; and some declined second-stage prosthetic implantation after infection control with a spacer in place, leading to permanent spacer retention.

Perioperative antimicrobial agents included vancomycin, meropenem, and voriconazole, and some patients also received local antibiotic administration postoperatively. Following discharge, most patients were prescribed oral levofloxacin and rifampin.

For patients with sinus tracts who underwent surgery, the sinus tracts were excised in their entirety, followed by primary closure of the skin opening. For cases with associated skin defects, local flap reconstruction was performed.

### 2.3. Variables

The variables collected in this study were as follows: age, sex, number of sinus tract openings, number of admissions, duration of the infection, hypertension, diabetes, coronary heart disease, arrhythmia, rheumatoid arthritis, psychiatric disorders, multiple PJIs, multiple arthroplasties, PJI sites, joint swelling or effusion, joint erythema and warmth, limited range of motion, local pain, preoperative microbiological specimen submission, treatment strategy, intraoperative specimens collected, intra-articular antibiotic administration, postoperative drainage, blood transfusion, cardiovascular complications, pulmonary complications, cerebrovascular complications, and PJI outcomes.

The exposure variable was the preoperative number of sinus tract openings. Patients without an opening were assigned a count of zero; separately documented external openings were counted individually. The number of sinus tract openings describes the external manifestation of the sinus tract, rather than the number of independent deep sinus tracts confirmed by surgical anatomy.

The endpoint was recorded as infection control, defined as no infection recurrence at the last documented clinical assessment. Records classified as not cured were analyzed as infection failures. Patients receiving antibiotic therapy alone or retaining a permanent spacer could be classified as having infection control. This endpoint does not establish antibiotic-free eradication or successful reimplantation [7,8].

### 2.4. Statistical Analysis

Continuous variables are summarized as mean ± standard deviation or median and interquartile range (IQR), and categorical variables as counts and percentages of observed records. Absolute standardized mean differences (SMDs) describe group imbalance. Between-group comparisons used Welch’s t-test for age, the Mann–Whitney U test for admissions and infection duration, and Fisher’s exact test for categorical variables. These nominal *p*-values describe baseline differences and were not used to select adjustment covariates. Infection duration was log-transformed as log(1 + weeks) when used in adjustment or balance assessment. Missing values were handled by complete-record analysis for each model; sample sizes and observed denominators are reported without imputation.

Firth bias-reduced logistic regression was used for the primary analysis because treatment failures and some treatment categories were sparse [9,10]. Infection control was the modeled outcome. The primary adjustment set comprised nominal treatment strategy and preoperative microbiological specimen submission, representing recorded management and diagnostic work-up. The model estimated eight slope parameters: one for opening count, six for the seven treatment categories, and one for specimen submission, plus the intercept. This corresponded to 2.9 failures per slope parameter. Covariates were not selected by univariate significance testing. Model complexity was assessed using the number of estimated parameters rather than variable labels; no fixed events-per-variable threshold was treated as proof of model adequacy [11].

Odds ratios and 95% confidence intervals (CIs) were obtained using profile penalized likelihood, with penalized likelihood-ratio tests. Conventional logistic regression with the same adjustment set was used for comparison. Sensitivity analyses varied the adjustment set; added age, sex, joint site, infection duration, or admission year; included intraoperative transfusion; restricted the analysis to operated patients; and excluded patients with five openings. An alternative clinical adjustment set included age, sex, joint site, and infection duration, with and without treatment strategy.

Secondary Firth models compared no opening, one opening, and multiple openings, with a two-degree-of-freedom global test. A model containing both sinus presence and numerical count and a separate analysis restricted to patients with openings examined count beyond binary presence. Joint-specific estimates and a count-by-joint interaction were assessed. 

For the separate comparison of any opening versus none, propensity scores included age, sex, joint site, log infection duration, and diabetes [12]. Patients were matched 1:1 without replacement using nearest neighbors, a caliper of 0.2 standard deviations of the logit propensity score, and exact matching on sex and joint site. Balance was assessed using SMDs with the prematching pooled standard deviations; outcomes were compared using an exact paired test and conditional OR.

Two-sided nominal *p*-values are reported with Holm and Bonferroni corrections across exploratory questions: numerical count, grouped exposure, count conditional on sinus presence, joint interaction, and matched sinus presence [13]. Analyses were performed in R 4.2.0.

## 3. Results

### 3.1. Patient Selection and Clinical Characteristics

**Figure 1:**
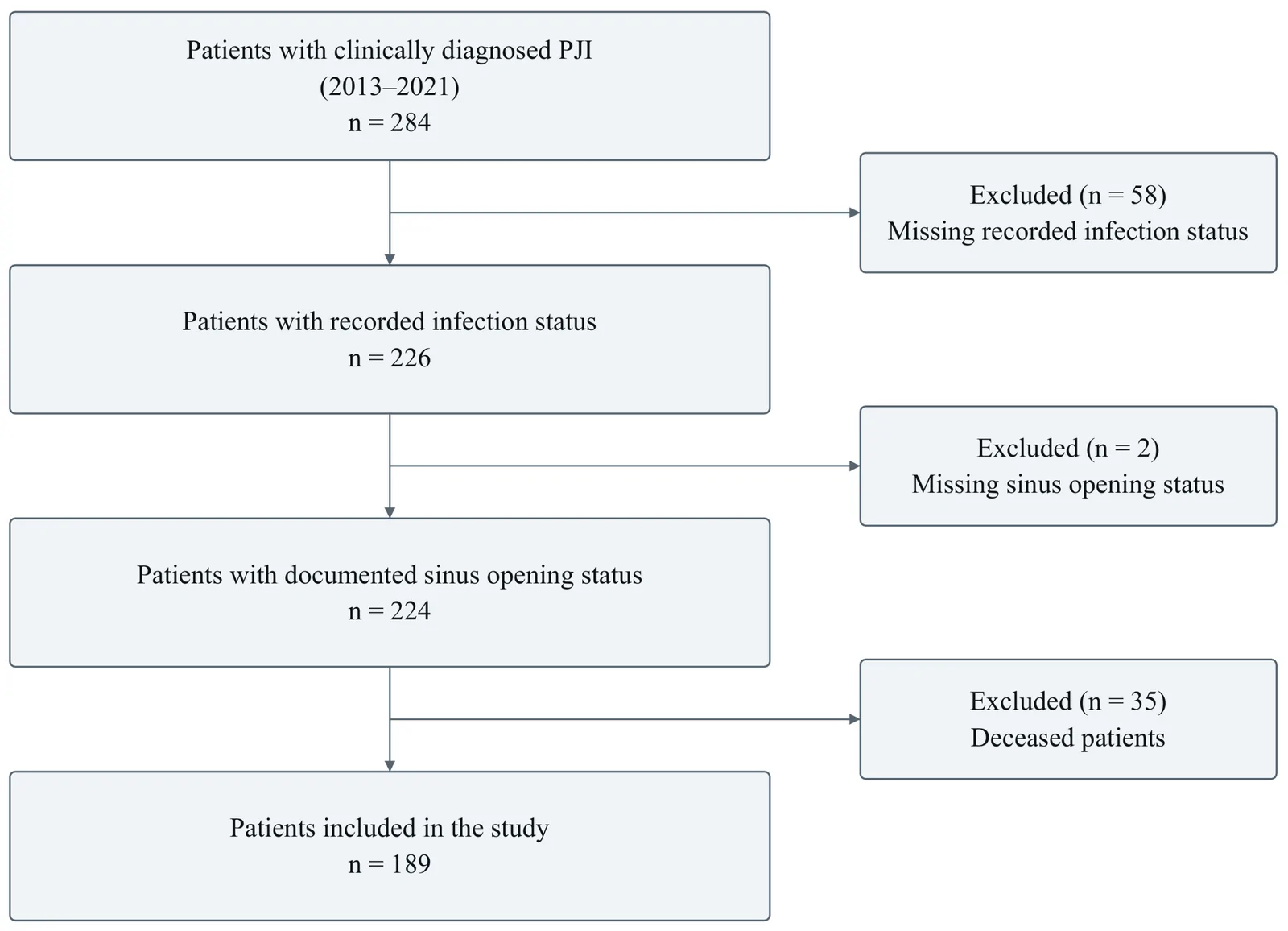
Selection of the study cohort.

Of the 284 available records of clinically diagnosed PJI in our digital system, 58 lacked follow-up outcome records, two further records lacked detailed information on sinus opening status, and another 35 records concerned deceased patients. Finally, there were 189 patients included in this study: 111 with knee PJI and 78 with hip PJI (Figure 1).

There were 116 patients without sinus tracts and 73 with at least one opening. Mean age was 65.2 ± 12.0 and 60.2 ± 11.8 years, respectively. Group differences included age, multiple PJIs, local inflammatory signs, preoperative specimen submission, and treatment strategy (Table 1 and Table 2). Two-stage revision was recorded in 19 patients without openings and none with openings, while permanent spacers were retained in 47 and 41 patients, respectively.

Infection control was recorded in 166 patients (87.8%), with 23 recorded failures (12.2%). Infection duration was missing in one patient, range of motion in two, preoperative specimen submission in one, treatment strategy in one, and intraoperative transfusion in 16. The primary adjusted analysis included 187 complete records.

### 3.2. Sinus Opening Distribution and Infection Control

**Figure 2:**
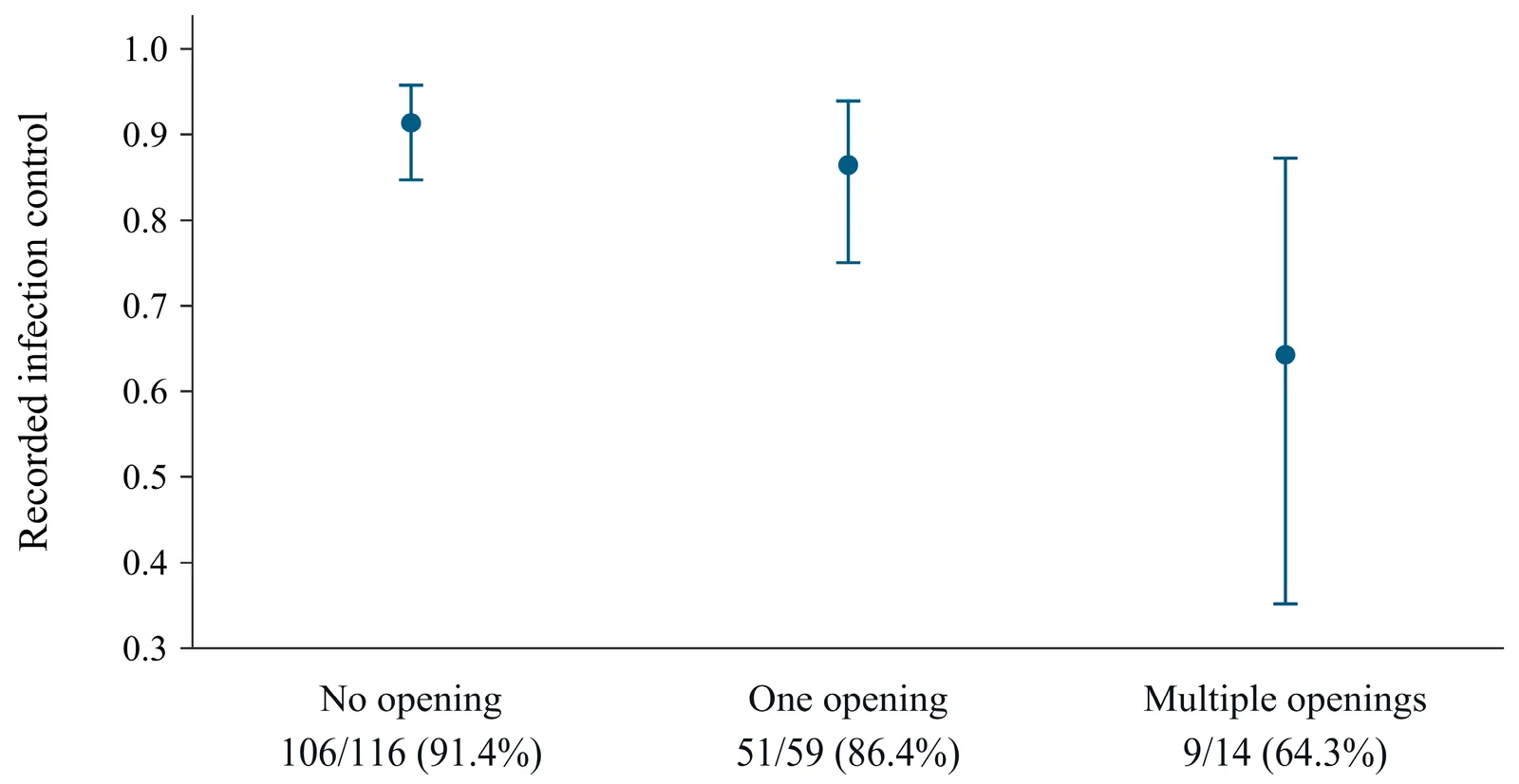
Recorded infection control proportions by sinus opening category. Error bars represent exact binomial 95% confidence intervals. The proportions refer to status at the last documented assessment.

Opening counts were one in 59, two in 9, three in 4, and five in 1. The mean among patients with openings was 1.29 ± 0.70. Fourteen patients had multiple openings. Infection control was recorded in 106/116 patients without openings (91.4%), 51/59 with one opening (86.4%), and 9/14 with multiple openings (64.3%). The complete distribution is shown in Table 3 and the grouped proportions with exact binomial confidence intervals are shown in Figure 2.

### 3.3. Association Between Opening Count and Infection Control

Higher opening count was associated with lower odds of infection control in the crude Firth model (OR 0.575, 95% CI 0.358–0.910; *p* = 0.0196) and the primary adjusted model (OR 0.562, 95% CI 0.340–0.912; *p* = 0.0209). Conventional adjusted logistic regression yielded a similar estimate (OR 0.543, 95% CI 0.325–0.906; *p* = 0.0195). In the grouped analysis, the adjusted ORs relative to no opening were 0.533 (95% CI 0.190–1.495; *p* = 0.2277) for one opening and 0.167 (95% CI 0.043–0.665; *p* = 0.0121) for multiple openings; the global *p*-value was 0.0384 (Table 4).

Sensitivity estimates remained in the same direction when adjustment was varied, the analysis was restricted to operated patients, or the patient with five openings was excluded (Table S1). The model additionally including infection duration yielded OR 0.623 (95% CI 0.378–1.020); the model adding intraoperative transfusion yielded OR 0.514 (95% CI 0.290–0.862; n = 172). Holm and Bonferroni adjustment each gave *p* = 0.1043 for the primary count association (Table S3).

### 3.4. Secondary and Matched Analyses

The numerical count estimate conditional on sinus presence was OR 0.614 (95% CI 0.300–1.250; *p* = 0.1626), while the estimate among patients with openings was OR 0.553 (95% CI 0.263–1.191; *p* = 0.1182). Joint-specific estimates were OR 0.531 (95% CI 0.267–0.972; *p* = 0.0407) for knee PJI and OR 0.679 (95% CI 0.313–1.498; *p* = 0.3229) for hip PJI, with no detected count-by-joint interaction (*p* = 0.9618), as shown in Table 4.

Propensity-score matching produced 60 pairs from 188 records with complete matching variables. All five absolute SMDs were below 0.10 after matching (Table S2). Infection control was recorded in 50/60 patients with openings and 56/60 without openings. The paired conditional OR was 0.333 (exact 95% CI 0.058–1.336; *p* = 0.1460). Multiplicity-adjusted results are summarized in Table S3.

## 4. Discussion

This study explored the relationship between a clinical sign in patients with PJI, the number of sinus tract openings, and recorded infection control after treatment: an increased number of preoperative openings may be associated with lower odds of recorded infection control. The proportions of patients with infection control in the no-opening, single-opening, and multiple-opening groups were 91.4%, 86.4%, and 64.3%, respectively. Estimates from conventional regression and the primary penalized regression model were similar. In addition, the direction of the association remained consistent across sensitivity analyses.

Through this study, we further characterized the local diversity of sinus tracts. We refined the preoperative sinus tract assessment from “presence or absence” to “number of openings” and found that an increased opening count was associated with lower odds of recorded infection control. The proportion of patients with infection control was 64.3% in the multiple-opening group, lower than 91.4% in the no-opening group; however, only 14 patients had multiple openings, and the primary association did not remain significant after Holm/Bonferroni correction (*p* = 0.1043). After propensity-score matching, the direction of the difference in infection control between patients with and without openings was consistent but not statistically significant (conditional OR 0.333, 95% CI 0.058–1.336, *p* = 0.1460). Given the limited matched sample size and number of events, definitive conclusions cannot be drawn from these results. The opening count provides a simple, objective candidate supplementary indicator for preoperative risk stratification, but its independent prognostic value requires prospective validation.

Previous studies provided a basis for further investigation of sinus tract characteristics. Xu et al. evaluated patients undergoing two-stage exchange arthroplasty and found that the presence of a sinus tract was associated with a higher likelihood of treatment failure; host factors and prior revision surgery were also associated with outcomes [3]. This suggests that sinus tracts should be interpreted within a more comprehensive clinical context. The present study extended the question from the presence or absence of a sinus tract to the number of openings and fully characterized the distribution of external openings.

In the DAIR study by Deng et al., the failure proportions in patients with and without a sinus tract were 36.5% and 27.3%, respectively, but the difference in survival comparison did not reach statistical significance [4]. This suggests that the prognostic significance of a sinus tract may depend on the study population, treatment setting, and outcome definition. Subsequently, Luger et al. reported that a sinus tract had high diagnostic specificity and was associated with lower 3-year infection-free survival [5]. These studies support continued attention to the clinical manifestations of sinus tracts, but they cannot be taken to indicate that sinus tracts have the same impact across all treatment strategies. Davis et al. examined this issue from another clinical perspective. Among patients with documented sinus tracts before definitive revision, 44% had previously received non-definitive management [14]. That study emphasized that a sinus tract should be regarded as part of the joint infection rather than an isolated superficial wound.

Gabrielli et al. studied 240 patients scheduled for two-stage exchange. A draining sinus was associated with failure to reach reimplantation, but the overall treatment-failure comparison was not statistically significant [15]. In a small case–control study of hip PJI, Xu et al. found that a sinus tract was more frequent in patients with reinfection after two-stage revision [16]. Completion of reimplantation and infection control are different outcomes. In our cohort, recorded infection control could include permanent spacer retention.

Previous studies established the basic understanding that the presence of a sinus tract is associated with poorer PJI prognosis, but most remained at the binary level of presence versus absence, and the findings were not entirely consistent. On this basis, the present study advanced from the presence or absence of a sinus tract to the number of sinus tract openings and described the distribution of sinus tract counts in patients with PJI. This offers a possible explanation for previous inconsistent findings: if single and multiple openings are combined as “any sinus tract,” the stronger signal associated with multiple openings may be diluted. A single opening may be relatively easier to completely excise and close with soft tissue, whereas multiple openings may indicate more extensive soft-tissue involvement and greater difficulty with debridement and coverage. In addition, this study reports joint-specific estimates and an interaction test for hip and knee PJI using opening count as the exposure; however, the joint-specific confidence intervals were wide and no interaction was detected (*p* = 0.9618). This does not establish equivalent associations in the two joints. Therefore, our study represents an exploratory assessment of opening count alongside sinus presence.

One possible explanation for the association between sinus tracts and recorded infection control is that multiple external openings may not merely represent an increase in number but may more likely reflect longer-standing sinus tract formation, a greater infection burden, and more extensive local soft-tissue involvement. Fibrosis, scarring, poor vascularity, and dead space may make debridement and soft-tissue coverage more difficult. These processes were not measured in this study. Even if intra-articular infection is addressed surgically, this adverse local environment may still increase the risk of infection-control failure. Therefore, multiple openings may be an external marker of infection severity and poor soft-tissue conditions rather than an independent causative factor. This interpretation is consistent with the view of Xu et al., who regarded sinus tracts as a marker of periarticular soft-tissue problems and prior surgical burden [3]. In 43 patients requiring gastrocnemius flap coverage for knee PJI, Theil et al. reported infection-free survival of 71% at two years and 63% at five years [17]. This cohort illustrates the difficulty of infection control in patients with major soft-tissue defects. That study did not evaluate opening count.

The findings of this study support recording both the presence or absence of a sinus tract and the number of openings during clinical evaluations. Compared with recording presence or absence alone, recording the number and location of openings can more clearly describe the external manifestation. This requires no additional laboratory tests or specialized imaging and can be completed during physical examination at admission. The number of openings should be used as part of a comprehensive assessment together with host status, prior surgical history, pathogens, soft-tissue conditions, and the planned treatment strategy, and should not be used alone as a basis for treatment decisions. For patients with multiple openings and poor local soft-tissue conditions, plastic surgery or soft-tissue reconstruction evaluation may be included in preoperative discussions to determine whether a flap or other coverage techniques are needed during revision [17]. These analyses did not test whether reconstructive procedures improve infection control.

This study focused on sinus tracts in patients with PJI, included both hip and knee PJI, and covered multiple treatment strategies recorded in routine clinical care. We reported every observed opening count, which helps clearly present the distribution of sinus tract opening counts. In addition, the grouped analysis provided clinically interpretable comparisons. Statistically, penalized regression helped address sparse-data bias. Sensitivity analyses, joint-site interaction testing, matched analysis, and multiple-testing correction evaluated the association from different perspectives.

This study also has several limitations. First, it was a single-center retrospective study with patient selection based on existing records. This limits the generalizability of the results and may leave residual confounding. Treatment modalities varied considerably and were related to clinical indications. Some treatment categories had limited overlap between patients with and without sinus tracts. Second, excluding deceased patients and records with missing outcomes may introduce selection bias. Follow-up duration and the timing and causes of death could not be verified. Opening counts were taken from routine clinical records, and the models did not adjust for specific pathogens. The number of recorded failures was small relative to the number of estimated parameters. Patients with multiple openings were also few. Therefore, even with penalized regression, estimation precision remained limited. Thus, multicenter prospective validation is needed before incorporating opening count into prognostic tools or treatment pathways.

## 5. Conclusions

In patients with PJI, an increased preoperative sinus tract opening count may be associated with lower odds of recorded infection control. Patients with multiple openings had a lower proportion of infection control; however, the sample size was small, the number of events was low, and the primary association was not statistically significant after multiple-testing correction. No interaction by joint site was detected. Future multicenter, prospective studies with standardized counting and uniform outcome definitions are needed to validate these findings and to assess whether incorporating opening count into preoperative risk stratification improves clinical decision-making and patient prognosis.

## Figures and Tables

**Table 1:** Baseline characteristics by sinus presence.

Characteristic	No Sinus Tract n = 116	Sinus Tract n = 73	Absolute SMD	*p*-Value
Age (years)	65.2 ± 12.0	60.2 ± 11.8	0.416	0.006
Female sex	72/116 (62.1%)	37/73 (50.7%)	0.230	0.133
Number of admissions	1.5 ± 0.8	1.5 ± 0.8	0.037	0.698
Hip PJI	42/116 (36.2%)	36/73 (49.3%)	0.266	0.095
Infection duration in weeks	17.0 (4.0–52.0)	17.0 (3.0–69.0)	0.059	0.665
Hypertension	46/116 (39.7%)	25/73 (34.2%)	0.112	0.538
Diabetes	16/116 (13.8%)	16/73 (21.9%)	0.212	0.166
Coronary heart disease	30/116 (25.9%)	9/73 (12.3%)	0.348	0.027
Arrhythmia	26/116 (22.4%)	17/73 (23.3%)	0.021	1.000
Rheumatoid arthritis	6/116 (5.2%)	2/73 (2.7%)	0.124	0.713
Psychiatric disorder	2/116 (1.7%)	1/73 (1.4%)	0.029	1.000
Multiple PJIs	1/116 (0.9%)	5/73 (6.8%)	0.313	0.033
Multiple arthroplasties	19/116 (16.4%)	10/73 (13.7%)	0.075	0.683
Joint swelling or effusion	83/116 (71.6%)	71/73 (97.3%)	0.754	<0.001
Erythema and warmth	73/116 (62.9%)	70/73 (95.9%)	0.888	<0.001
Limited range of motion	114/116 (98.3%)	71/71 (100.0%)	0.187	0.526
Local pain or tenderness	105/116 (90.5%)	73/73 (100.0%)	0.456	0.007

Values are mean ± standard deviation, median (IQR), or n/observed N (%). SMD, standardized mean difference; IQR, interquartile range. Infection-duration SMD uses log(1 + weeks). Duration was missing in one patient and range of motion in two. Two-sided nominal *p*-values use Welch’s t-test (age), Mann–Whitney U tests (admissions and duration), or Fisher’s exact tests (categorical variables). SMD describes imbalance and has no corresponding *p*-value.

**Table 2:** Preoperative microbiological specimen submission and treatment.

Diagnostic Work-Up or Treatment	No Opening n = 116	Any Opening n = 73
Preoperative specimen submission for microbiological testing	56/116 (48.3%)	49/72 (68.1%)
Antibiotics without surgery	10/116 (8.6%)	5/72 (6.9%)
One-stage revision	14/116 (12.1%)	5/72 (6.9%)
Two-stage revision	19/116 (16.4%)	0/72 (0.0%)
DAIR	15/116 (12.9%)	8/72 (11.1%)
Debridement or irrigation	11/116 (9.5%)	12/72 (16.7%)
Amputation	0/116 (0.0%)	1/72 (1.4%)
Permanent spacer retention	47/116 (40.5%)	41/72 (56.9%)

Values are n/observed N (%). Preoperative specimen submission and treatment strategy were each missing in one patient. DAIR: debridement, antibiotics, and implant retention. Spacer retention was permanent.

**Table 3:** Sinus opening count and recorded infection control.

Sinus Opening Count	Patients	Infection Control	Infection Failure	Control Proportion
0	116	106	10	91.4%
1	59	51	8	86.4%
2	9	6	3	66.7%
3	4	2	2	50.0%
5	1	1	0	100.0%
All	189	166	23	87.8%

Infection control denotes no recurrence at the last documented assessment. Multiple openings were present in 14 patients, of whom nine had infection control and five had failure.

**Table 4:** Associations between sinus opening count and recorded infection control.

Analysis	Patients	OR (95% CI)	*p*-Value
Crude count: Firth regression	189	0.575 (0.358–0.910)	0.0196
Adjusted count: Firth regression	187	0.562 (0.340–0.912)	0.0209
Adjusted count: logistic regression	187	0.543 (0.325–0.906)	0.0195
One opening versus none	187	0.533 (0.190–1.495)	0.2277
Multiple openings versus none	187	0.167 (0.043–0.665)	0.0121
Count with sinus presence in model	187	0.614 (0.300–1.250)	0.1626
Count among patients with openings	71	0.553 (0.263–1.191)	0.1182
Knee PJI: count association	110	0.531 (0.267–0.972)	0.0407
Hip PJI: count association	77	0.679 (0.313–1.498)	0.3229
Hip versus knee: ratio of count ORs	187	1.023 (0.399–2.707)	0.9618

The primary adjustment set comprised treatment strategy and preoperative microbiological specimen submission; the model estimated eight slope parameters plus the intercept. Firth estimates use profile penalized-likelihood confidence intervals and likelihood-ratio tests; the conventional estimate uses Wald inference. Grouped contrasts use no opening as the reference; global *p* = 0.0384. Subgroup models retain the same adjustment variables where estimable. *p*-values are unadjusted; multiplicity corrections appear in Table S3. CI, confidence interval; OR, odds ratio.

## Data Availability

The clinical dataset contains information derived from patient records and is not publicly posted. Requests for deidentified data may be directed to the corresponding author, subject to institutional approval and applicable privacy restrictions.

## Supplementary Materials

The following supporting information can be downloaded at https://www.mdpi.com/article/10.3390/jcm15218702/s1. Table S1: Sensitivity analyses; Table S2: Propensity-score balance and matched comparison; Table S3: Multiplicity-adjusted tests. These tables are appended for review.
