# Supplementary material for: The Sinus Tract Opening Count and Recorded Infection Control in Hip and Knee Prosthetic Joint Infection
Source: J Clin Med. 2026 Nov 7;15(21):8702. doi: 10.3390/jcm15218702 (PMC13764483; doi:10.3390/jcm15218702)
Supplement: Supplementary file 1 [file jcm-15-08702-s001.zip › jcm-4605620-supplementary.pdf]

Supplementary Tables

Table S1. Sensitivity analyses

Analysis	Patients	OR (95% CI)	p-value
Treatment adjustment only	188	0.552 (0.335–0.893)	0.0169
Specimen-submission adjustment only	188	0.587 (0.364–0.935)	0.0261
Additional age, sex, and joint site	187	0.569 (0.347–0.919)	0.0223
Additional infection duration	186	0.623 (0.378–1.020)	0.0593
Additional admission year	187	0.562 (0.329–0.924)	0.0237
Operated patients only	172	0.536 (0.308–0.895)	0.0183
Excluding the patient with five openings	186	0.456 (0.256–0.801)	0.0068
Additional intraoperative transfusion: Firth	172	0.514 (0.290–0.862)	0.0127
Additional intraoperative transfusion: conventional	172	0.484 (0.275–0.855)	0.0124
Clinical baseline adjustment	188	0.625 (0.387–0.996)	0.0479
Clinical baseline adjustment plus treatment	187	0.621 (0.379–1.008)	0.0539

Unless otherwise specified, sensitivity analyses modify the primary model adjusted for treatment strategy and preoperative microbiological specimen submission. Clinical baseline adjustment comprises age, sex, joint site, and log(1 + infection duration in weeks); the treatment extension adds nominal treatment strategy. All estimates use Firth regression except the conventional transfusion model. Sample sizes reflect complete records for each model. Confidence intervals and p-values are unadjusted.

Table S2. Propensity-score balance and matched comparison

**Matching variable**	**Absolute SMD before**	**Absolute SMD after**
Age	0.413	0.004
Sex	0.223	0.000
Joint site	0.259	0.000
Log infection duration	0.059	0.066
Diabetes	0.209	0.043

SMD, standardized mean difference. Matching used age, sex, joint site, log infection duration, and diabetes. Sixty pairs were obtained from 188 complete records. Infection control occurred in 50/60 patients with openings and 56/60 without openings. The paired conditional OR was 0.333 (exact 95% CI 0.058–1.336; p = 0.1460).

Table S3. Multiplicity-adjusted tests

**Analytical question**	**Unadjusted p**	**Holm p**	**Bonferroni p**
Numerical opening count	0.0209	0.1043	0.1043
Grouped opening categories: global test	0.0384	0.1536	0.1920
Count conditional on sinus presence	0.1626	0.4380	0.8128
Count-by-joint interaction	0.9618	0.9618	1.0000
Matched any opening versus none	0.1460	0.4380	0.7300

The five-question family comprises the primary count association, grouped global exposure, count conditional on sinus presence, count-by-joint interaction, and matched sinus presence. Regression tests use the Firth adjustment set; matching uses an exact paired test. Grouped exposure has two degrees of freedom; other regression tests have one. Corrections are exploratory; confidence intervals in other tables are unadjusted.
